# Severe Community-Acquired Pneumonia Caused by Human Adenovirus in Immunocompetent Adults: A Multicenter Case Series

**DOI:** 10.1371/journal.pone.0151199

**Published:** 2016-03-11

**Authors:** Dingyu Tan, Huadong Zhu, Yangyang Fu, Fei Tong, Dongqi Yao, Joseph Walline, Jun Xu, Xuezhong Yu

**Affiliations:** 1 Department of Emergency, Peking Union Medical College Hospital, Chinese Academy of Medical sciences, Beijing, China; 2 Department of Emergency, Second Hospital of Hebei Medical University, Shijiazhuang, Hebei province, China; 3 Division of Emergency Medicine, Department of Surgery, Saint Louis University Hospital, Saint Louis, Missouri, United States of America; University Hospital San Giovanni Battista di Torino, ITALY

## Abstract

**Background:**

Severe community-acquired pneumonia (CAP) caused by human adenovirus (HAdV), especially HAdV type 55 (HAdV-55) in immunocompetent adults has raised increasing concerns. Clinical knowledge of severe CAP and acute respiratory distress syndrome induced by HAdV-55 is still limited, though the pathogen has been fully characterized by whole-genome sequencing.

**Methods:**

We conducted a multicentre retrospective review of all consecutive patients with severe CAP caused by HAdV in immunocompetent adults admitted to the Emergency Department Intensive Care Unit of two hospitals in Northern China between February 2012 and April 2014. Clinical, laboratory, radiological characteristics, treatments and outcomes of these patients were collected and analyzed.

**Results:**

A total of 15 consecutive severe CAP patients with laboratory-confirmed adenovirus infections were included. The median age was 30 years and all cases were identified during the winter and spring seasons. HAdV-55 was the most frequently (11/15) detected HAdV type. Persistent high fever, cough and rapid progression of dyspnea were typically reported in these patients. Significantly increased pneumonia severity index (PSI), respiratory rate, and lower PaO_2_/FiO_2_, hypersensitive CRP were reported in non-survivors compared to survivors (P = 0.013, 0.022, 0.019 and 0.026, respectively). The rapid development of bilateral consolidations within 10 days after illness onset were the most common radiographic finding, usually accompanied by adjacent ground glass opacities and pleural effusions. Total mortality was 26.7% in this study. Corticosteroids were prescribed to 14 patients in this report, but the utilization rate between survivors and non-survivors was not significant.

**Conclusions:**

HAdV and the HAdV-55 sub-type play an important role among viral pneumonia pathogens in hospitalized immunocompetent adults in Northern China. HAdV should be tested in severe CAP patients with negative bacterial cultures and a lack of response to antibiotic treatment, even if radiologic imaging and clinical presentation initially suggest bacterial pneumonia.

## Introduction

Community-acquired pneumonia (CAP) is the leading infectious cause of death worldwide. Respiratory viruses, whose pathogen role have been underestimated for many years, account for more than 22% of adult CAP cases [1, 2]. Human adenoviruses (HAdVs) are non-enveloped, double-stranded DNA viruses, ubiquitous in the environment, and are also a common cause of respiratory infections. HAdV pneumonia typically is limited to newborns, immunocompromised hosts, and school or military camp populations [3]. Severe HAdV pneumonia has been frequently described in immunocompromised patients. Respiratory infection caused by HAdV in immunocompetent patients is usually thought to be mild and self-limited. However, with advances in modern molecular techniques, HAdV has been increasingly found to be involved in sporadic cases and outbreaks of severe CAP in healthy adults [4, 5].

To date, at least 69 HAdV genotypes have been recognized, and they are assigned to seven subgroups (A–G) according to genetic, biophysical and biochemical characteristics [6]. Species B (HAdV-3, -7, -11, and -14) is the predominant HAdV specie associated with respiratory infections among adults. HAdV-55, a genotype recombination between HAdV-11 and HAdV-14, has been the reported cause of sporadic or outbreak events of CAP in Turkey [7], Singapore [8], Korea [9] and China from 2004. HAdV-55-related sequential outbreaks in several provinces and regions of China gained increased attention since 2006 [10–12]. As just one pathogen of CAP, HAdV accounts for 5% of adult CAP cases in northern China between 2010 and 2012, while HAdV-55 accounts for more than 40% of identified HAdV positive samples [2]. Comparative studies showed that the serotypes of HAdV may be one of the factors which determine the severity of adenoviral pneumonia. HAdV-55 was thought to be more closely associated with severe pneumonia as compared to other serotypes (HAdV-3, -7 and -14) [2]. Clinical understanding of severe CAP induced by HAdV-55 is still limited, though the pathogen has been fully characterized by whole-genome sequencing. Current knowledge of HAdV-55-induced severe pneumonia is mainly derived from single case reports or relatively small, single-center series.

Beginning in February 2012, virus screening was carried out for an etiological study of adult severe CAP at the Peking Union Medical College Hospital (PUMCH) in Beijing and the Second Hospital of Hebei Medical University (SHHMU) in Hebei province, China. These two institutions, which have high referral rate in Northern China, encounter multiple cases of HAdV pneumonia annually. The present multicenter case series report describes in detail the clinical features and outcomes of severe HAdV CAP in immunocompetent adults.

## Materials and Methods

### Study population

The institutional ethics review boards of PUMCH and SHHMU approved this retrospective study. All data on the study patients was analyzed anonymously. The requirement for informed consent by individual patients was waived given the retrospective nature of the study. We performed a multicentre retrospective review of all consecutive patients diagnosed with severe HAdV CAP in the Emergency Department Intensive Care Unit (ICU) of the two hospitals between February 1, 2012 and April 30, 2014. The criteria for severe CAP was defined by modified American Thoracic Society criteria [13] or risk class V of the Pneumonia Severity Index (PSI) [14]. HAdV infection was confirmed by positive multiplex polymerase chain reaction (PCR) for HAdV from lower respiratory tract samples, such as sputum, tracheal aspirates or bronchoalveolar lavage (BAL) fluid. Additionally, we required negative other microbiological testing including bacterial, fungal or other common respiratory viruses at admission. Patients with HIV infection or neutropenia, those receiving immunosuppressive chemotherapy, pregnant or breast-feeding women, and those with malignant neoplasms were excluded.

### Microbiological evaluation

CAP patients whose clinical conditions deteriorated rapidly despite appropriate use of broad-spectrum antibiotics for 2–3 days were suspected of having atypical pneumonia. Lower respiratory tract samples, urine, blood, and acute and convalescent serum samples were collected for thorough microbiological tests including: (1) sputum specimens for staining and microbiological cultures; (2) urine specimens for *Legionella pneumophila* and *S*. *pneumonia* antigen detection; (3) blood cultures; (4) lower respiratory tract samples for virus real-time PCR detection (tested viruses included: HAdV, rhinovirus, cytomegalovirus, influenza virus, metapneumovirus, parainfluenza virus, coronavirus, and respiratory syncytial virus); (5) acute and convalescent serum samples for *Chlamydia Pneumoniae*, *Mycoplasma pneumoniae* and *Legionella pneumophila* antibody titer determination.

### Detection of HAdV and other respiratory viruses

Lower respiratory tract specimens of all patients were sent to the Institute of Medical Biology of the Chinese Academy of Medical Sciences, to test for respiratory viruses. Viral nucleic acids were extracted from each specimen using the NucliSens easyMAG system (bioMérieux, Marcy l’Etoile, France). Multiplex real-time PCR was performed to screen for HAdV, rhinovirus, cytomegalovirus, influenza virus, metapneumovirus, parainfluenza virus, coronavirus, and respiratory syncytial virus, using a Seeplex RV 15 ACE Detection kit (Seegene, Inc, Seoul, Korea) according to the manufacturer’s instructions. Specimens that were positive for HAdV were subjected to further analysis to determine the type of HAdV.

### Determination of HAdV types

HAdV positive specimens were subjected to a multiplex PCR assay for species with species-specific primers. Then, PCR targeting the hexon gene was performed again with type-specific primers. The PCR products were purified (Qiagen, Valencia, CA, USA), and then sequenced using the 3130xl Genetic Analyzer (Applied Biosystems, Foster City, CA, USA). Types of HAdV were confirmed by nucleotide blast analysis using the BLASTn program (National Center for Biotechnology Information, Bethesda, MD, USA).

### Clinical data collection

Investigators created a standardized data form for clinical information collection that included demographics (age, sex and smoking status), PSI score (to assess the severity of illness at admission), comorbidities, clinical symptoms (e.g. fever, chills, cough, sputum, dyspnea, chest pain, diarrhea, myalgias and sore throat), vital signs, laboratory tests, arterial blood gas analysis results, microbiological findings (including HAdV types) and chest radiologic characteristics. Concomitant medications, respiratory support, complications (e.g. use of mechanical ventilation or septic shock) and overall outcomes were also recorded.

### Statistical analysis

Continuous variables are expressed as median (interquartile range) and categorical variables as number and percent. Differences between groups were assessed with the Fisher exact test for categorical variables and the Mann-Whitney U test for continuous variables. Significance was fixed at *P*<0.05.

## Results

A total 15 cases (seven from SHHMU and eight from PUMCH) were confirmed with HAdV as the only pathogen of pneumonia. Two patients with HAdV positive samples were excluded due to possible coinfection with *Legionella pneumophila* and *S*. *pneumonia* respectively. All 15 patients were admitted to the ICU and fulfilled the criteria of severe CAP and of acute respiratory distress syndrome (ARDS).

### Demographics and HAdV typing

**Fig 1:**
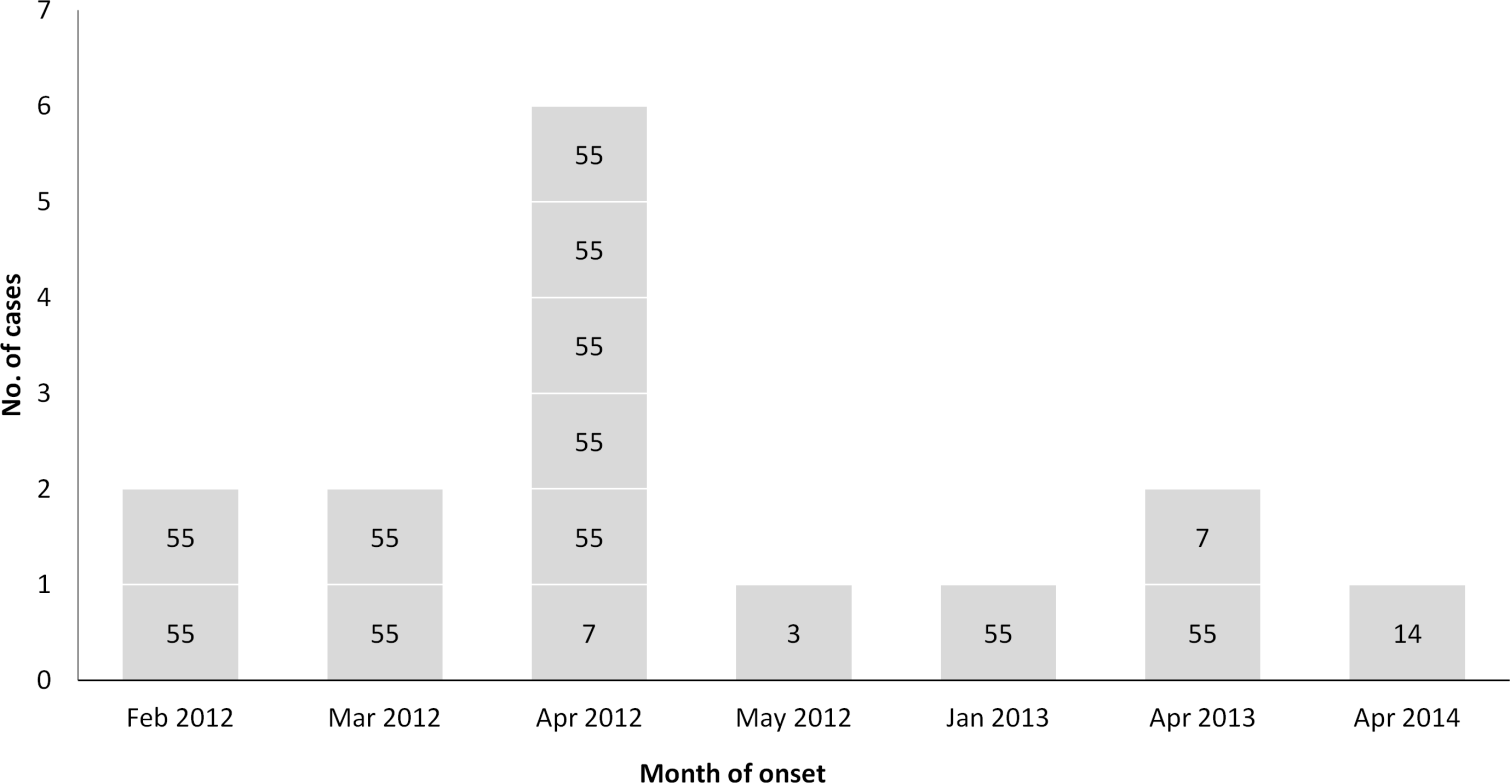
Epidemiologic distribution of different types of human adenoviruses. Most cases of human adenovirus type 55 were identified in April.

Patients with severe HAdV CAP usually were young and middle-aged adults and the median age was 30 years (range, 18 to 52 years). Male gender (73.3%) was more common than female gender, and all patients who did not survive were male. All 15 patients were non-immunocompromised. Three patients (who were aged >50) had underlying diseases of hypertension. Seven patients were smokers. Most patients (14 of 15 patients) came from the Beijing-Tianjin-Hebei Region of Northern China. The illnesses of all patients occurred in the winter and spring seasons, and most HAdVs (60%) were identified in April. Definitive HAdV typing of the 15 cases were all classed as species B (11 were type 55, two were type 7, one was type 3, and one was type 14) (see Fig 1).

### Clinical characteristics: Comparison Between survivors and non-survivors

As shown in Table 1, flu-like symptoms, such as fever, cough and little sputum, were most commonly observed at the onset of illness. All patients presented with a high fever, with a median body temperature of 39.8°C (range, 39.0°C to 40.8°C), which persisted for 7 days (range, 1 to 10 days). Most clinical symptoms and signs between survivors and non-survivors did not differ, except for PSI score and respiratory rate; these were significantly higher in non-survivors (P = 0.013 and 0.022, respectively). The median time from onset to ICU admission was seven days, and most patients (86.7%) showed apparent dyspnea at admission. Arterial blood gas analysis at ICU admission revealed profound hypoxia, with a median PaO_2_/FiO_2_ of 120 mmHg. PaO_2_/FiO_2_ in non-survivors was significantly lower than that in survivors (p = 0.019). There was no difference in laboratory findings between survivors and non-survivors, except that hypersensitive CRP in non-survivors were significantly lower than in survivors (P = 0.026) (Table 2). White blood cell counts in most patients were in the normal range. Serum aspartate aminotransferase (AST), lactate dehydrogenase (LDH), creatinine phosphokinase (CK), creatinine and urea nitrogen levels were higher in non-survivors, but the differences were not significant.

**Table 1:** Demographic and clinical characteristics and outcomes of patients with severe CAP caused by Adenoviruses (comparison between survivors and non-survivors).

Characteristics	All cases (n = 15)	Survivors (n = 11)	Non-survivors (n = 4)	*P* Value
Age, years	30(22–38)	30(23–38)	28(19.3–48)	0.744
Male sex, %	11(73.3)	7(63.6)	4(100)	0.516
Underlying diseases	3(26.7)	2(18.2)	1(50)	1.0
Smoking history	7(46.7)	6(54.5)	1(25)	0.569
Clinical features				
Duration before admission, days	7 (6–10)	7 (6–8)	8.5(4.8–10)	0.640
Initial PSI score	95(68–116)	84(62–98)	127.5(103.3–175)	0.013
Duration before identification of HAdV, days	11(10–13)	11(10–13)	12.5(9.8–13.8)	0.510
HAdV-55	11(73.3)	8(72.8)	3(75)	1.0
Tmax, °C	39.8(39.5–40.0)	39.8(39.5–40.0)	39.9(39.2–40.0)	0.790
Cough	15(100)	11(100)	4(100)	1.0
Purulent sputum	6(40.0)	6(54.5)	0(0)	0.103
Bloody sputum	5(33.3)	5(45.5)	0(0)	0.231
Dyspnea	13(86.7)	10(90.9)	3(75)	1.476
Dyspnea before admission, days	3(1.5–4)	3(1.8–4.3)	3(1–4)	0.793
Chest pain	3(20.0)	3(27.3)	0(0)	0.516
Diarrhea	3(20.0)	2(18.2)	1(25.0)	1.0
Myalgia	3(20.0)	3(27.3)	0(0)	0.516
Sore throat	2(13.3)	1(9.1)	1(25.0)	0.476
Chill	6(40.0)	4(36.4)	2(50.0)	1.0
Initial vital signs				
Heart rate, beats/min	108(95–120)	101(90–118)	126.5(100.5–157.8)	0.170
Respiratory rate, breaths/min	26(21–30)	24(21–27)	33(27.3–46.3)	0.022
MAP, mm Hg	80.7(75.3–92.7)	80.7(75.3–92.3)	94.7(55.2–121.9)	0.472
PaO_**2**_ /FiO_**2**_ ratio, mm Hg	120(75–162)	152(97.1–200)	71(50.3–85.8)	0.019
Coinfection during hospitalization	7(46.7)	5(45.5)	2(40)	1.0
Management				
Antiviral	8(53.3)	5(45.5)	3(75)	0.569
Adjuvant IVIG	10(66.7)	7(63.6)	3(75)	1.0
Corticosteroids	14(93.3)	10(90.9)	4(100)	1.0
Mechanical ventilation (MV)^a^	14(93.3)	10(90.9)	4(100)	1.0
Need for vasopressor	6(40)	3(27.3)	3(75)	0.560
Outcomes				
Length of ICU stay, days	11(6–19)	14(7–19)	4.5(3.3–18.5)	0.116
Duration of MV, days	7.5(4.8–13.3)	8.5(5.8–13.3)	4.5(3.3–18.5)	0.356

CAP, community-acquired pneumonia; PSI, pneumonia severity index; HAdV, human adenovirus; MAP, mean arterial pressure; IVIG, intravenous immunoglobulin.

^a^One patient in survivors received non-invasive ventilation.

**Table 2:** Laboratory Findings and Chest Radiologic Characteristics of Patients with severe CAP caused by Adenoviruses (comparison between survivors and non-survivors).

Characteristic	All cases (n = 15)	Surviors (n = 11)	Non-surviors (n = 4)	*P* Value
Laboratory Findings				
WBC, 10^**9**^ /L	7.2(5. 0–10.5)	7.3(6.1–10.6)	4.1 (1.3–9.1)	0.117
Neutrophil, %	85.3 (80.3–88.9)	84.3 (80.3–87)	93.1 (71.5–95.1)	0.151
Lymphocyte, %	10.1(7.3–12)	10.2(8.1–12.0)	6.2(4.5–24.3)	0.192
Hematocrit, %	38.4(34.4–41.1)	38.4(30.7–40.4)	39.8(37.7–44.6)	0.327
Platelet, 10^**9**^ /L	134(97–169)	134(87–158)	135.5(102–265.8)	0.433
hsCRP, mg/dL	88.0(48.4–157.6)	121.73(67.0–175.4)	36.62(15.0–78.1)	0.026
AST, U /L	143(29–165)	143(24–162)	200(33.8–490)	0.433
ALT, U /L	63 (19–98)	63(19–94)	78(22.5–366)	0.556
ALB, g/L	29.3(24.5–32.9)	31(24.8–32.9)	26.9(24.13–32.2	0.695
LDH, U /L	340(326–1074)	338(279–1057)	1032.5(370.8–1805.3)	0.151
CK, U /L	111(94–840)	100(684–408)	1344(116.3–2164.5)	0.090
Tbil, mmol/L	16.4(11.9–35.4)	16.4(12–28.2)	23.3(11.1–70.8)	0.896
Cr, umol/L	80(59.2–123)	77 (52.5–95)	114.8(67.6–137.7)	0.240
Bun, mmol/L	5.0(3.4–6.0)	4.2(3.4–5.8)	9.0(5.1–11.7)	0.068
Na, mmol/L	131(130–132)	132(130–132)	131(130–132)	0.179
Chest radiography				
Pleural effusions	11(73.3)	8(72.7)	3(75)	1.0
Diffuse ground-glass opacities	2(13.3)	2(18.2)	0(0)	1.0
Bilateral Consolidations	13(86.7)	9(81.8)	4(100)	1.0
No. of lobes involved ≥4	6(40)	2(18.2)	4(100)	0.011
Consolidation with ground glass opacities	8(53.3)	7(63.6)	1(25)	0.282

CAP, community-acquired pneumonia; WBC, white blood cell; hsCRP, hypersensitive C reactive protein; ALT, alanine aminotransferase; AST, aspartate aminotransferase; ALB, albumin; LDH, lactate dehydrogenase; CK, creatine kinase; Tbil, total bilirubin; Cr, creatinine; Bun, blood urea nitrogen.

### Radiographic features

**Fig 2:**
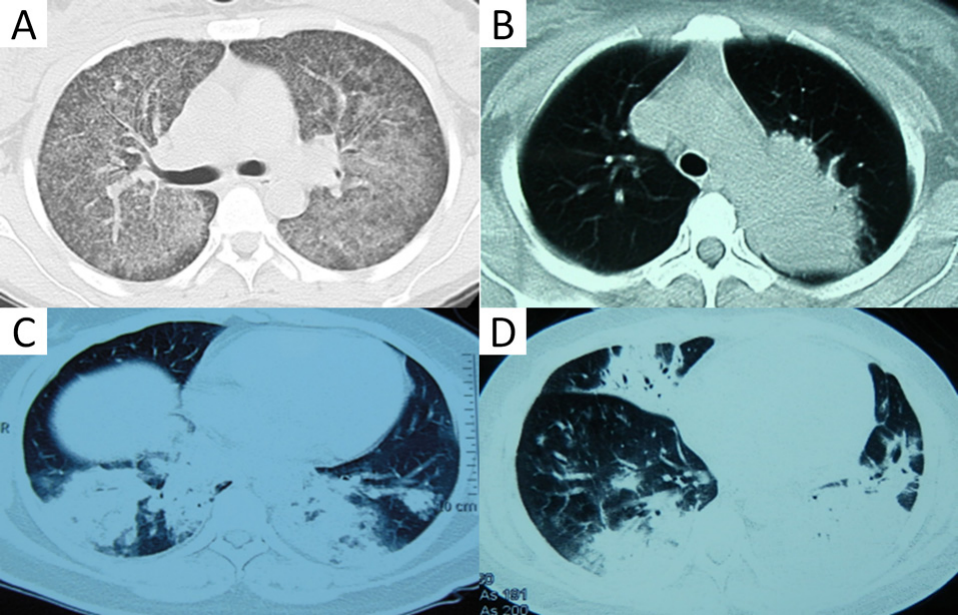
Chest CT findings in patients with severe human adenoviruses pneumonia. (A), 30 year-old woman with adenovirus type 55 pneumonia. Initial chest CT scan (day 6 after the onset of illness) shows bilateral diffuse ground-grass opacities. (B), 29 year-old woman with adenovirus type 55 pneumonia. Chest CT scan five days after the onset shows consolidation in the left upper lobe. (C), 38 year-old woman with adenovirus type 14 pneumonia. Initial CT scan obtained on day 7 shows apparent consolidation with slight patchy ground-glass opacities in both lower lungs. (D), 22 year-old man with adenovirus type 7 pneumonia. CT scan obtained on day 10 shows bilateral consolidations accompanied by adjacent ground glass opacities and pleural effusions.

**Fig 3:**
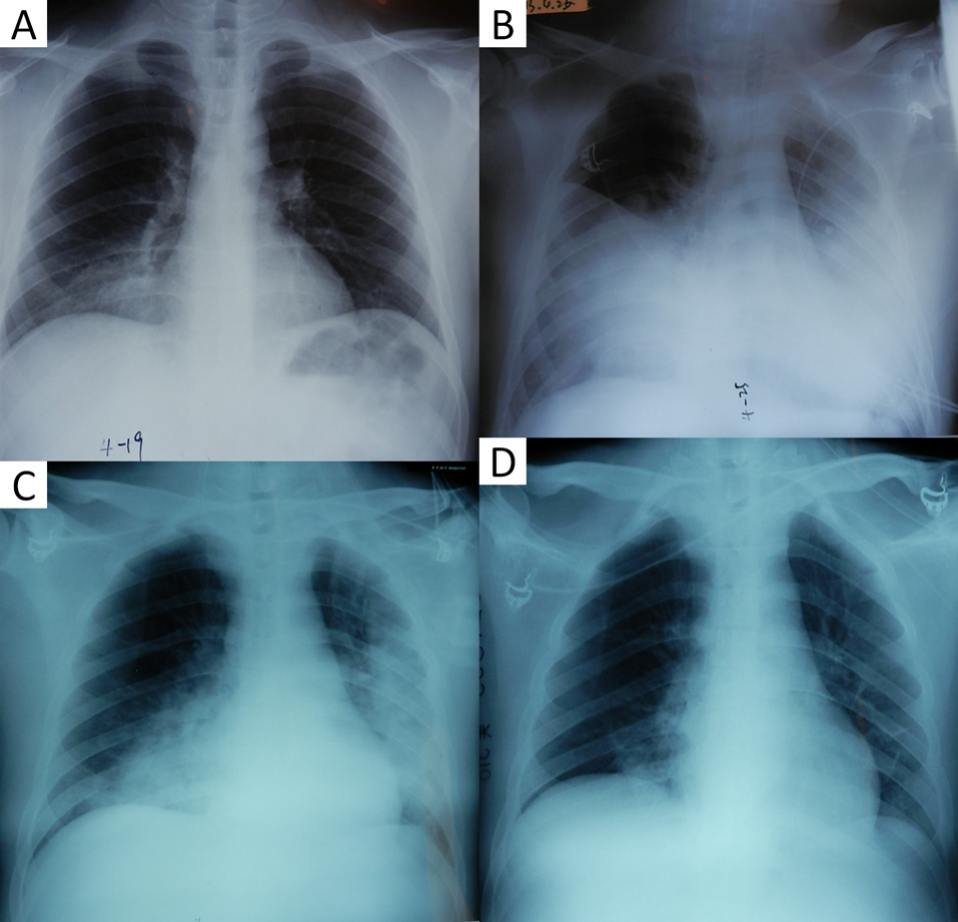
23 year-old man with adenovirus type 55 pneumonia. (A), Initial chest radiograph (day 2 after the onset of illness) shows focal consolidation in the right lower lobe. (B), Chest radiograph (day 8 after the onset of illness) reveals rapid progression, widespread consolidations in both lungs. (C), Consolidations in both lungs have begun to decrease by day 15 after onset. (D), Consolidations in both lungs have further decreased by day 18 after onset.

Consolidation (86.7%), ground-grass opacities (GGOs) (66.7%), and pleural effusions (73.3%) were the most common findings in severe HAdV CAP (Table 2). Diffuse GGOs were revealed as predominant in two patients (13.3%) (Fig 2A). Within one week after illness onset, focal consolidation was the predominant finding in ten patients (Figs 2B and 3A), while three patients showed bilateral multiple consolidations accompanied by GGOs in two patients (Fig 2C). The most commonly involved lobe was the right lower lobe (n = 5), followed by the left lower lobe (n = 3), left upper lobe (n = 1), and right upper lobe (n = 1). Follow-up radiologic findings in 13 patients with consolidation showed the rapid development of bilateral consolidations within 10 days after illness onset, usually accompanied by adjacent GGOs and pleural effusions (Fig 2D and S1 Fig). The number of lobes with consolidation was significantly increased in non-survivors than in survivors (p = 0.011). The parenchymal abnormalities began to absorb about two weeks after illness onset, with no appearances of fibrosis.

### Treatment and outcome

Overall treatment outcomes of study patients are summarized in Table 2. Empirical broad-spectrum intravenous antibiotics were given to all the patients, while eight were prescribed antiviral drugs. Antiviral treatment was started prior to the confirmation of viral pneumonia in our study. Acylovir, gancyclovir, oseltamivir and ribavirin are all widely used empirically in China. In our study, oseltamivir was used in all eight patients who received antiviral drugs, while ribavirin and gancyclovir were used in combination with oseltamivir in four and three patients, respectively. Ten out of 15 (66.7%) patients received adjuvant intravenous immunoglobulin for 3–5 days, while fourteen (93.3%) received corticosteroids for 3–10 days, including hydrocortisone or methylprednisolone. Hospital acquired pulmonary infections developed in seven cases, and *Acinetobacter baumannii* was the dominating pathogen. Four patients did not survive (26.7%). One case died of respiratory failure caused by ARDS and three other patients died of multiple organ dysfunction syndrome which mainly included respiratory failure and septic shock. The need for mechanical ventilation and/or vasopressors were similar between non-survivors and survivors. Length of ICU stay and duration of mechanical ventilation in non-survivors were less than that in survivors, but the differences were not significant.

## Discussion

In the past decade, adult CAP induced by HAdV has been causing increased concern worldwide, especially in China. HAdV accounted for 0.8% of adult acute respiratory tract infections in Beijing between 2005 and 2010, while HAdV-3 was the most frequently detected serotype [15]. However, the proportion of HAdV CAP increased to 4.2%-5% in the following two years [2, 16], and HAdV-55 became the predominant culprit. HAdV-55 is thought to be closely associated with severe CAP, and the results of our study verified this phenomenon again. To the best of our knowledge, the number of patients needed mechanical ventilation (14 patients) in this report is significantly more than those of two to six cases in all recent investigations of HAdV-55 related severe CAP. Our study may provide some new understanding for clinical characteristics of HAdV-55 related severe CAP in immunocompetent adults.

Demographics in our report were consistent with the previous investigations [17, 18]. Young and middle-aged male adults were susceptible hosts for severe HAdV CAP. All patients of severe HAdV CAP occurred in the winter and spring seasons just after the influenza epidemic, which indicates that the relationship between HAdV CAP and influenza may be an appropriate focus for future research. All severe CAP caused by HAdV-55 in immunocompetent adults in China in previous investigations occurred in Northern China, mainly in Baoding, Hebei province and Beijing. The wider dispersion of our cases suggests that HAdV-55 is still active in Northern China, and might be expanding its scope of influence.

The clinical features and disease progression of our cases are highly similar to those of ARDS caused by HAdV-55 or other types of HAdV described in previous reports [19]. All patients presented with high fevers and cough. Common manifestations of HAdV infection such as diarrhea or conjunctivitis were not common in our patients. PSI scores, one of the most commonly used severity assessment tools for CAP, can also provide important prognostic information for HAdV-55 induced CAP [2]. In this report, absence of underlying diseases and abnormal laboratory findings such as decreased hematocrit, hyponatremia and hyperglycemia, highlight the contribution of abnormal respiratory rate and PaO_2_/FiO_2_ to the high values we saw in patients’ PSI scores. Respiratory rate is an independent risk marker for in-hospital mortality of CAP [20]. Low CRP values are commonly considered to be associated with a less severe systemic inflammatory response, which should reflect a better prognosis. Interestingly, our data showed that among patients with ARDS caused by HAdV, a low initial serum CRP level at admission correlated to a fatal outcome. Interpretation of this result may be difficult, though a similar phenomenon was found in severe CAP caused by *Streptococcus pneumoniae* [21] and CAP patients with liver cirrhosis [22]. CRP is an acute phase protein, which is synthesized by the liver in response to tissue damage. It may be reasonable to speculate that severe HAdV CAP accompanied by slightly elevated CRP may indicate impairment of the protective inflammatory response.

In accordance with the reputation of respiratory viruses for causing diffuse interstitial infiltrates, Clark reported bilateral GGOs as the most common abnormality in 22 immunocompetent adults of adenovirus pneumonia [4]. However, more and more research supports consolidation rather than interstitial infiltrates as the main imaging characteristic of adenovirus pneumonia in immunocompetent adults. To date, adenovirus is the only virus known to cause focal or lobar consolidation (the typical manifestation of bacterial pneumonia) as its main imaging abnormality. The initial image findings of adenovirus pneumonia, which resemble bacterial pneumonia may mislead diagnosis and treatment. As Gu concluded, single lobar or segmental consolidation was more common in patients without ARDS, while patients with ARDS had bilateral and multilobar lung consolidations [17]. Compared to survivors in this report, non-survivors had more extensive consolidations which correlated with respiratory rate and PaO_2_ /FiO_2_.

At present there is no specific treatment for severe HAdV pneumonia and high mortality rates are often reported in cases of severe HAdV pneumonia. Among patients needing mechanical ventilation in previous reports about severe HAdV-55 pneumonia, nine out of 22 (40.9%) cases died [2, 9, 17–19]. Early administration of cidofovir showed possible effect in six immunocompetent patients [9], but randomized controlled trials are lacking and cidofovir is not available in most hospitals in China. Mortality (26.7%) in our study is relatively low and corticosteroids were frequently used in this study. Adjuvant corticosteroids for CAP and ARDS remains controversial. Improved mortality and a good safety record for corticosteroids given to patients with severe CAP were seen by some meta-analyses [23–25]. However, corticosteroids have also been found to be an independent predictor of mortality or other poor outcomes in multiple studies of other viral pneumonias [26–28]. The effect of corticosteroids on severe HAdV CAP is still uncertain, although there have been a few case reports. One case of a fatal HAdV-3 pneumonia with hypercytokinemia, “pulse” methylprednisolone treatment resulted in the rapid amelioration of the patient’s respiratory distress [29]. In a study focused on severe CAP caused by HAdV-55, 10 out of 18 patients received corticosteroids and total mortality was 5.9% [17]. However, given the small sample size and design of our non-controlled study, we can not conclude that corticosteroids are helpful in severe HAdV CAP.

Our study has several limitations. First, for the determined HAdV types (especially type 55), whole-genome sequencings were not performed due to its retrospective nature. Second, there were a relatively small number of cases because of the relatively low incidence of severe adenovirus pneumonia. The results of comparison between survivors and non-survivors may also lead to bias due to the unequal number of patients in each group. Finally, treatment effects of cidofovir and other antiviral drugs could not be compared because cidofovir was not available in the two hospitals in this study.

In conclusion, HAdV-55 plays a very important role in cases of adenovirus pneumonia in immunocompetent adults of Northern China, and surveillance of this agent as a cause of severe CAP is warranted. HAdV should be tested in severe CAP patients with initial negative bacterial cultures and a failure to respond to antibiotics, even though image findings and clinical presentation might suggest bacterial pneumonia.

## Supporting Information

S1 Fig 50 year-old man with adenovirus type 55 pneumonia. (A), Initial CT scan obtained at the level of tracheal bifurcation on day 5 after the onset of illness shows focal consolidation in the left lower lung. (B), Rapid progression of consolidations in both lung zones with emerging patchy ground-glass opacities by day 8 after onset. (C), Consolidations in both lungs have obviously decreased on day 16 after onset, with residual patchy ground-glass opacities. (D), Parenchymal abnormalities have further decreased in both lungs by day 21 after onset with residual consolidation in the left lower lobe.
(TIF)
