# Supplementary figures and images for: Severe Community-Acquired Pneumonia Caused by Human Adenovirus in Immunocompetent Adults: A Multicenter Case Series
Source: PLoS One. 2016 Mar 11;11(3):e0151199. doi: 10.1371/journal.pone.0151199 (PMC4788423; doi:10.1371/journal.pone.0151199)

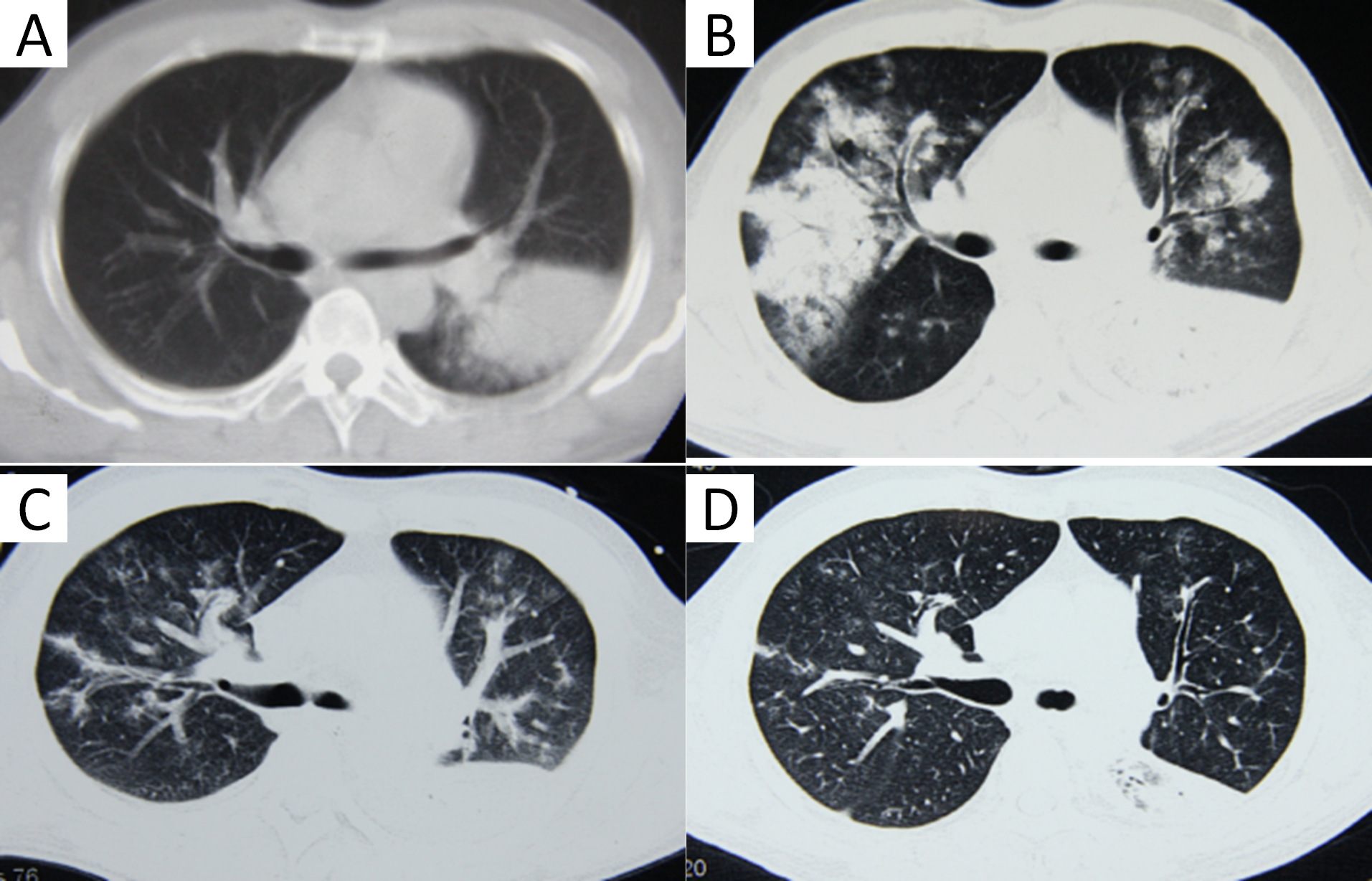

Supplement: S1 Fig — (A), Initial CT scan obtained at the level of tracheal bifurcation on day 5 after the onset of illness shows focal consolidation in the left lower lung. (B), Rapid progression of consolidations in both lung zones with emerging patchy ground-glass opacities by day 8 after onset. (C), Consolidations in both lungs have obviously decreased on day 16 after onset, with residual patchy ground-glass opacities. (D), Parenchymal abnormalities have further decreased in both lungs by day 21 after onset with residual consolidation in the left lower lobe. (TIF) [file pone.0151199.s001.tif]
